# Genetic Consequences of Forest Fragmentation for a Highly Specialized Arboreal Mammal - the Edible Dormouse

**DOI:** 10.1371/journal.pone.0088092

**Published:** 2014-02-04

**Authors:** Joanna Fietz, Jürgen Tomiuk, Volker Loeschcke, Tanja Weis-Dootz, Gernot Segelbacher

**Affiliations:** 1 Animal Husbandry and Animal Breeding, University of Hohenheim, Stuttgart, Germany; 2 Department of Biosciences, Integrative Ecology and Evolution, Aarhus University, Aarhus, Denmark; 3 Institute of Experimental Ecology, University of Ulm, Ulm, Germany; 4 Wildlife Ecology and Management, University Freiburg, Freiburg, Germany; Institute of Evolutionary Biology (CSIC-UPF), Spain

## Abstract

Habitat loss and fragmentation represent the most serious extinction threats for many species and have been demonstrated to be especially detrimental for mammals. Particularly, highly specialized species with low dispersal abilities will encounter a high risk of extinction in fragmented landscapes. Here we studied the edible dormouse (*Glis glis*), a small arboreal mammal that is distributed throughout Central Europe, where forests are mostly fragmented at different spatial scales. The aim of this study was to investigate the effect of habitat fragmentation on genetic population structures using the example of edible dormouse populations inhabiting forest fragments in south western Germany. We genotyped 380 adult individuals captured between 2001 and 2009 in four different forest fragments and one large continuous forest using 14 species-specific microsatellites. We hypothesised, that populations in small forest patches have a lower genetic diversity and are more isolated compared to populations living in continuous forests. In accordance with our expectations we found that dormice inhabiting forest fragments were isolated from each other. Furthermore, their genetic population structure was more unstable over the study period than in the large continuous forest. Even though we could not detect lower genetic variability within individuals inhabiting forest fragments, strong genetic isolation and an overall high risk to mate with close relatives might be precursors to a reduced genetic variability and the onset of inbreeding depression. Results of this study highlight that connectivity among habitat fragments can already be strongly hampered before genetic erosion within small and isolated populations becomes evident.

## Introduction

Habitat loss, degradation and fragmentation represent the most serious extinction threats for mammals, affecting 40% of the species assessed [1]. Among the different ecosystems, forests are the ones that are most strongly suffering from worldwide human impact, not only due to exploitation for timber but also to clear cutting for agriculture [2]. Such an ongoing habitat loss and fragmentation leads to the continuous isolation of suitable habitat patches across landscapes. Arboreal mammals with low dispersal ability thus face the problem that they become increasingly subdivided into comparatively small and isolated populations [3], which are subject to genetic drift and inbreeding, eventually resulting in a loss of genetic variation [4]; [5]; [6]. Such forest habitat specialists can therefore be considered as especially vulnerable to habitat fragmentation as the decrease in the level of genetic variation, inbreeding and the concomitant inbreeding effects can be expected to reduce their individual fitness and to obstruct adaptive responses to environmental stressful conditions [7]. Accordingly, arboreal species, common ringtail possums (*Pseudocheirus peregrinus*) and Australian squirrel gliders (*Petaurus norfolcensis*), were shown to display lower levels of allelic richness and observed heterozygosity in small isolated populations compared to populations inhabiting larger habitat patches [5]; [6].

Edible dormice (*Glis glis*; rodentia) are extreme habitat specialists, occurring preferentially in deciduous mixed forests dominated by European beech (*Fagus sylvatica*) and oaks (*Quercus spec.*) [8]; [9]. The life history of this small arboreal mammal is tightly linked to the seed production of its main feeding tree species [10]; [11]; [12], as it depends on the energy rich seeds during pre-hibernation fattening and when raising its offspring [13]. Thus edible dormice depend on mature deciduous forests for food and also for tree holes. Monitoring studies carried out in Italy showed that this habitat specialist responds particularly sensitive to increasing isolation and decreasing size of forest patches [9]; [14]; [15]; [16]; [17]. The highest probability of detecting edible dormice was associated with a patch size of 40 ha and more [15]. In Germany, on the other hand, edible dormice were shown to occur quite frequently within small forest fragments (<30 ha) [18]; [19] and even in higher population densities than found in populations inhabiting large forests. Differences in the presence of dormice within small forest fragments between Italy and Germany might be explained by habitat quality (maturity of the forest) and the intensity of forest management [15]; [17]. Results of capture-mark-recapture and radio tracking studies further showed that edible dormice avoid to cross streets and open ground (e.g. meadows and ski-pistes) [20]; [21]; [22], and only cross potential barriers if they can move from branch to branch [21]. However, in contrast to these findings two capture-mark-recapture studies recorded dispersal movements across open ground, even though only 2.5% and less of all marked individuals were observed to do so (5 out of 196 and 18 out of 1,042) [18]; [23]. In these populations high population densities between 20 and 57 individuals/ha and poor habitat quality of the study patches, such as shrubs and hedges, might have forced individuals to emigrate. To simulate such high-motivation situations, Negro et al. 2013 [22] performed experiments with edible dormice, translocating them from one to another forest which were separated by a ski-piste of 80 m width. Even dormice never crossed these barriers spontaneously, 37% (four out of 11) of these translocated individuals were able to cross the ski-piste and came back to their original forest patch. This shows that edible dormice are able to cross open ground but do it rarely deliberately.

In accordance with patterns described by the island rule for small mammals, edible dormice inhabiting forest fragments were shown to be significantly larger and heavier than individuals in the continuous forest and occurred also in higher numbers [19]. The same pattern could also be found in different species of tree squirrels (*Sciurus carolinensis*, *S. niger* and *Tamiasciurus hudsonicus*) [24].

Edible dormice provide an excellent model to study effects of forest fragmentation and potential isolation effects on the genetic and demographic structure in animal populations. They are extreme habitat specialist, have low dispersal abilities and were shown to be particularly sensitive to habitat fragmentation. We here characterised the genetic structure and variability of dormice inhabiting a large continuous forest and several comparatively small forest fragments. Results of our study highlight that connectivity among habitat fragments can already be strongly hampered before genetic erosion within small and isolated populations becomes evident.

## Materials and Methods

### Species

The arboreal edible dormouse (*G. glis*) is the largest European dormouse with a body mass between 80 and 120 g. In Central Europe, this nocturnal rodent occurs preferentially in deciduous mixed forests dominated by the European beech (*F. sylvatica*) [8]. Edible dormice are obligate hibernators and in Germany, adults hibernate in underground burrows for 7–8 months, generally from September until June. During hibernation, they cease feeding and rely entirely on stored fat accumulated during the previous autumn for energy metabolism. Characteristically for fat-storing hibernators, body mass dramatically increases shortly before the onset of the hibernation period [13]. When raising their offspring and fattening for hibernation, dormice feed predominantly on beech nuts and acorns, but also consume leaves, buds, and fruits [13]. They reach sexual maturity after their first hibernation period as yearlings. In Germany they produce only one litter per year and mate between the end of June and July. After a gestation period of about 30 days, litters averaging 5–6 young are born [25]; [26], that are raised by their mothers without paternal help. In this small mammal reproduction is synchronized to the presence or absence of beechnuts and/or acorns. In years with a lack of seeding beech trees, dormice typically stay sexually inactive, and dramatically reduced or even absent reproduction under these conditions was observed independently in several free-living dormouse populations [10]; [11]; [12]. Thus the number of juveniles born varies extremely among years depending on the seeding pattern of beeches (*F. sylvatica*) and oaks (*Quercus spec.*). Reproductive activity seems to be energetically costly, not only for females but also for male edible dormice [27]; [28] and a former study revealed low survival rates of both sexes in years with high reproduction [29]. For a small mammal, edible dormice are comparatively long-lived and may live up to 11 years with an average lifespan of 3–4 years in the wild [29]. Dormice frequently use nest boxes to rest during the day and to rear their offspring, which makes the access to the animals for scientific studies comparatively easy. In Germany, edible dormice are classified as strictly protected (*Regulation of Wildlife Conservation in the Federal Republic of Germany*). However, in the state of Baden-Württemberg, they are not listed as endangered [8] and occur in medium to high population densities within old mixed deciduous forests, even within small forest fragments [8]; [19]. Our studies were conducted under licence from the Nature Conservancy (Permit Number: 55-6/8852.15) and the Committee on the Ethics of Animal Experiments of the Regional Commission of Tübingen (Permit Number: 892).

### Sampling

**Figure 1:**
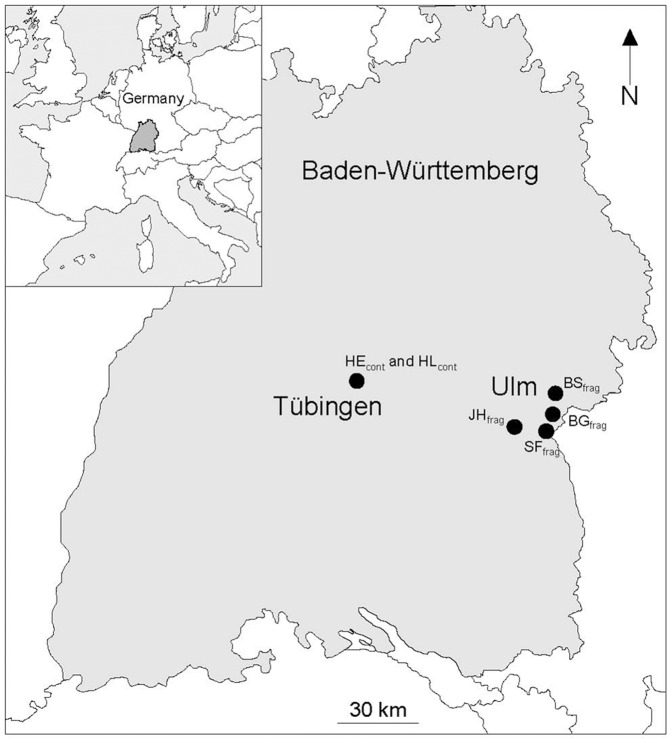
Map of Baden-Württemberg (Germany) showing the positions of the study sites at two locations. See Table 1 for detailed information. Insert shows the map of Europe with Baden-Württemberg in dark grey.

**Figure 2:**
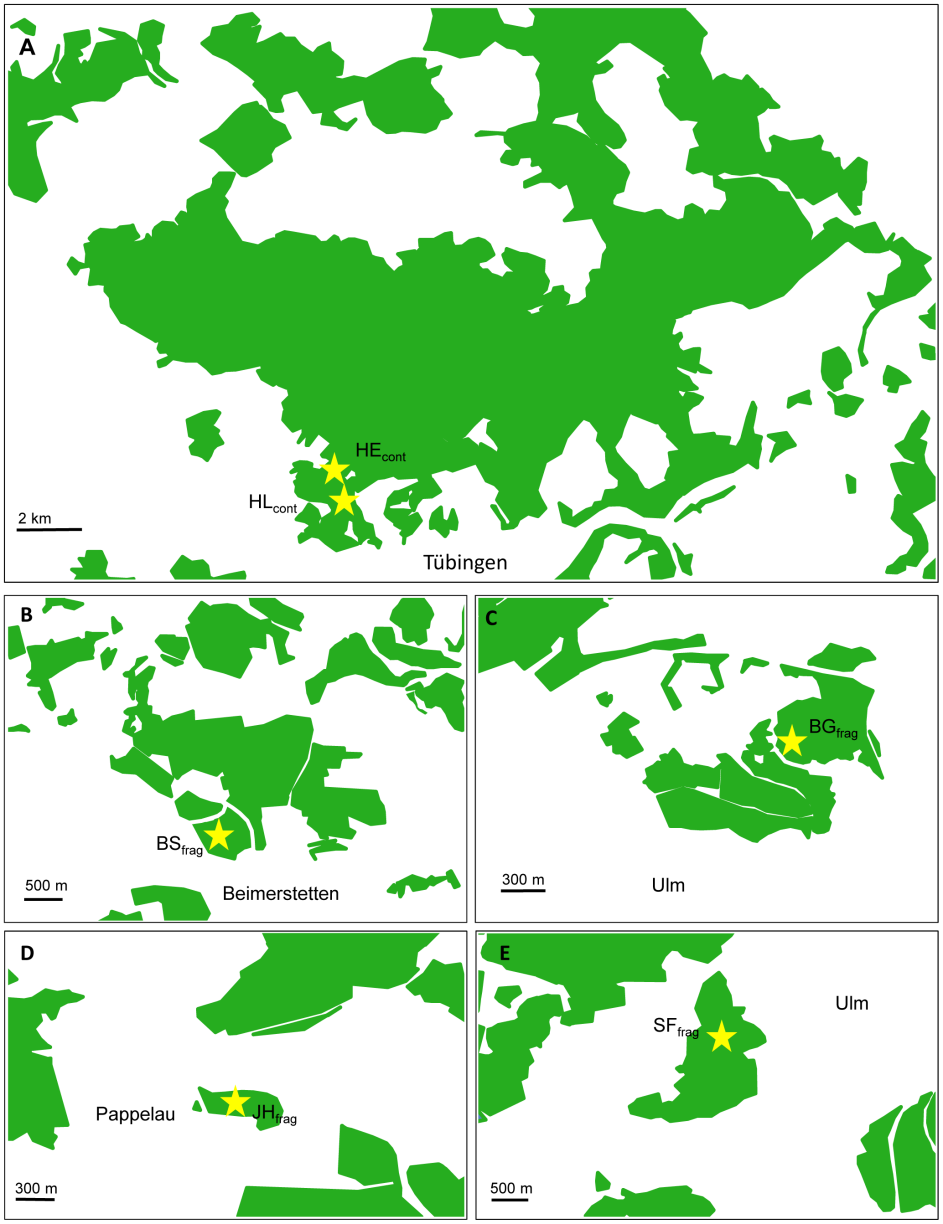
Maps showing: A) study sites HE_cont_ and HL_cont_ within the continuous forest close to the town Tübingen; B–E) study sites within the forest fragments close to the town Ulm (B: BS_frag_; C: BG_frag_; D: JH_frag_; E: SF_frag_). Green areas mark forest cover and asterisks the centre of the respective study sites.

This study was carried out at six different sites located all in mature beech (*F. sylvatica*) dominated mixed deciduous forests in south western Germany (Table 1, Fig. 1 & Fig. 2). The first two study sites (HE_cont_ and HL_cont_) located 0.7 km apart from each other (Table 2, Fig. 2A), were situated close to the town Tübingen on the southern rim of the “Schönbuch” Nature Reserve [30], which represents one of the largest continuous forest areas within the state of Baden-Württemberg. The other four study sites were located within forest fragments of different sizes close to the town Ulm which is located 70 km away from Tübingen (Table 1, Fig. 1 & Fig. 2B–E). These four forest fragments are surrounded by agricultural fields and meadows, are in part also limited by tarmac roads and railway trails, and they adjoin cities and villages. In a former systematic capture-mark-recapture study carried out at these study sites, we showed that tarmac roads and pastures with minimal widths of about 15 m represent effective migration barriers for edible dormice [31]. The study site BS_frag_ adjoins at its eastern side a monoculture of spruce (*Picea abies*) with an extension of about 300 m. At the eastern border of this monoculture runs a railway trail from north to south, encompassed by pasture (width 15 to 60 m). In the north the forest adjoins a pasture with 35 m width. In the south the nearest forest fragment is in about 500 m distance (Fig. 2B). The forest fragment of BG_frag_ is limited by tarmac roads at the eastern and northern borders (width: 15 m and 50 m, respectively; Fig. 2C) and adjoins the town Ulm in the south. The nearest forest fragments in the south west and in the east of BG_frag_ have distances of about 1.5 km. JH_frag_ is completely surrounded by fields with the nearest forest in about 400 m distance. A tarmac and a gravel road are running at its western side (Fig. 2D), with the small village Pappelau located on the other side of the road. Also SF_frag_ is completely surrounded by fields and meadows. The nearest forest fragment is located 125 m apart in the north of the forest fragment (Fig. 2E). None of these forest fragments is connected by continuous hedgerows with the surrounding forests (Fig. 2B–E). All of these forest fragments have been isolated since the 1880s (pers. comm. R. Lemm; regional forest administration Ulm). Assuming an average lifespan of 3–4 years in edible dormice [29], we argue that these forest fragments have been isolated for approximately 50 dormouse generations. The distances among these fragments range from 4 to 17 km (Table 1 & Table 2). In each study site, nest boxes were installed three meters above ground at the intersections of grid lines marking 30×30 m squares.

**Table 2:** Geographic distances (km) between study sites.

study site abbreviations	HL_cont_	BS_frag_	BG_frag_	JH_frag_	SF_frag_
HE_cont_	0.7	72	72	62	71
HL_cont_		72	72	62	71
BS_frag_			7	17	12
BG_frag_				12	4
JH_frag_					8

**Table 1:** Study sites of dormouse populations: abbreviations, locations, coordinates, type and size of forests and study sites, period, number of nest boxes and genotyped individuals; population density: number of individuals (excluding juveniles) captured per hectare; population size: assumed number of individuals inhabiting the respective forest (forest size [ha] × population density [ind./ha]).

study site abbreviations	locations	coordinates	forest type	forest size [ha]	size of study site [ha]	period of data collection	number of nest boxes	number of genotyped individuals	population density (individuals/ha)	population size
HE_cont_	Tübingen	48°33′03.63″N8°59′55.63″E	continuous	15.000	12	2001–2009	128	149	5.4^a^	72,750
HL_cont_	Tübingen	48°32′26.86″N9°0′15.28″E	continuous	15.000	8.4	2001–2009	80	52	4.3^a^	72,750
BS_frag_	Ulm	48°29′21.84″N9°58′14.60″E	fragment	33	7	2007–2009	70	45	10.8^b^	356
BG_frag_	Ulm	48°25′19.12″N9°57′43.56″E	fragment	70	7	2006–2009	70	70	24.0^b^	1,680
JH_frag_	Ulm	48°22′10.84″N9°49′04.06″E	fragment	11	7	2009	70	10	3.3	36
SF_frag_	Ulm	48°23′12.98″N9°55′55.49″E	fragment	135	7	2007–2009	70	54	10.8^b^	1,458

a 29;

b 19.

We checked the nest boxes regularly from June to October during daytime (resting period) between 2001 and 2009 (Table 1). Upon first capture, we marked individuals with transponders (Trovan, EURO I.D. Usling, Weilerswist, Germany) and recorded sex and age. During each field season, individuals were classified as adults if they had overwintered at least once. Skin samples (1×2 mm) were collected from each of 380 adult individuals (184 females and 196 males). Samples were taken from ear lobes, conserved in 70% ethanol and stored at −20°C. Sample sizes for our genetic studies varied considerably among years and sites (Table S1 in File S1).

Edible dormice show high site fidelity [25]; [29]. Therefore, we assumed that individuals that were not recaptured within a certain year, but captured both in earlier and later years, had been present within the study site between these capture years. These individuals were accounted for within the yearly genotype samples, resulting in a total of 654 genotypes when analysing genotypic variation among years.

### Genetic analyses

DNA was extracted with a QIAamp Kit (Qiagen, Hilden, Germany), following the manufacturer's instructions. For genotyping we used a total of 14 microsatellites. We used 11 microsatellites (Table S2 in File S1: Glis173, Glis196, Glis223, Glis228, Glis239, Glis243, Glis365, Glis376, Glis426, Glis483 and Glis 487) for which we designed species-specific primers [32] and three microsatellites (5pilch, 8pilch and 11pilch) for which primers and PCR-conditions were described in another study on edible dormice [33]. PCR amplifications of the first set of 11 microsatellites were performed in a 10 µl volume consisting of 1× QIAGEN PCR buffer, 0.025 mM of each primer, 3 mM MgCl_2_, 0.40 mM of each dNTP and 0.5 U Taq DNA Polymerase (Qiagen) and 1 µl template using a Gradient Cycler PTC-200 (Biozym Diagnostik, Germany). A touchdown thermal cycling programme encompassing a 10°C span of annealing temperatures ranging between 60°C and 50°C was used for amplification. Following an initial denaturation step of 95°C for 3 min, cycling parameters were 20 cycles at 95°C for 30 s, 60°C annealing temperature (decreased 0.5°C per cycle) for 30 s and 72°C for 40 s and 15 cycles of 95°C for 30 s, 50°C for 30 s and 72°C for 40 s and a final extension step of 72°C for 5 min. All PCR-products were run on an ABI 310 sequencer (Applied Biosystems, California, USA) and their fragment length was determined with the programme *Genescan* v3.7 (Applied Biosystems 2001).

### Statistical analyses of genetic data

The statistics of genetic data was done in three steps. First, the basic analyses consider allele and genotype frequencies within and among populations in order to disclose effects on individual loci, e.g. selection or assortative mating, which can change the genetic structure of populations. Furthermore, an elementary prerequisite of subsequent analyses is to use a set of unlinked loci, thus we tested pair wise loci for their genotypic disequilibrium.

Second, we were interested to detect the sources of genetic variability of populations in south western Germany, i.e. the total genetic variation is partitioned into distinct fractions which originate from differences among populations and locations but also from deviations from random mating (AMOVA). We further tested whether the genetic population structures at the location Ulm can be explained by isolation by distance (MANTEL test). Beyond that effects of forest fragmentation on the population structure are of interest. The intention of our study was to show that populations living in forest fragments are strictly isolated having each their characteristic genetic population background whereas populations living in continuous forests share a common gene pool (STRUCTURE).

Third, we graphically demonstrate our hitherto existing statistical results by using traditional cluster analyses, i.e. we apply a simple cluster procedure to group populations (UPGMA) with respect to the genetic distances between pairs of populations (NEI's distance). This graphically shows the temporal and spatial similarities within populations across years and differences among populations within different locations.

#### Genetic variation of individual loci

The software programme *Genepop* version 4.1.3 [34] was used to estimate allele frequencies as well as observed and expected degrees of heterozygosity. The degree of heterozygosity averaged over the total study period is a weighed mean using yearly sample sizes.

Derived from the degree of homozygosity we also obtained the effective number of alleles. *Genepop* was also used to test for deviations of observed genotype frequencies from Hardy-Weinberg proportion (HWE), the genotypic linkage disequilibrium between pairs of loci in the samples of each year, and finally the allelic and genotypic homogeneity among populations, and within and among populations sampled in different years. The homogeneity tests performed by *Genepop* are exact tests and in case of large sample sizes combined with Monte-Carlo-procedures. The basic level of significance α was set to 0.05 and, in the case of multiple testing we applied a sequential Bonferroni correction compensating for the risk of an inflating type 1 error.

The allelic richness (AR) in populations was estimated using the programme *Fstat* version 2.8 [35], [36]. Allelic richness is standardized to the smallest number of individuals sampled from the population within the study period (Tübingen - HE_cont_: N = 11; HL_cont_: N = 9; Ulm – BG_frag_: N = 10; BS_frag_: N = 12; SF_frag_: N = 14; JH_frag_: N = 11). This measure takes into account sample size [37] and thus can be used to compare samples from different populations.

The genotypic patterns of locus Glis483 indicated sex-linked inheritance as in males only homozygotes were observed, whereas in females also heterozygotes could be found. Consequently, in cases of testing Hardy-Weinberg proportion male genotypes at this locus were not considered. The appearance of stutter bands hindered a reliable fragment length analysis of the di-nucleotide microsatellite 11pilch and, therefore, we used a 4bp-allelic raster to minimize the misclassification of genotypes (Table S3 in File S1).

#### Genetic variability within and among populations

We considered different genetic sources that can explain the genetic variation of populations and which might be different in the populations of the continuous forest and the forest fragments, i.e. genetic differences between locations (Tübingen and Ulm), among populations of the two locations (Tübingen: HE_cont_ and HL_cont_; Ulm: BG_frag_, BS_frag_, SF_frag_ and JH_frag_), among yearly samples of populations, among individuals within populations, and within individuals.

The sources of genetic variance were determined using the software *Arlequin* version 3.5.1.2 [38]. The procedure AMOVA estimates also population specific F_IS_-values which estimate deviations from HWE. For this purpose all individuals sampled in the total study period are considered representative for the respective subpopulations and genetic data of recaptured individuals are used only once.

The procedure AMOVA was applied separately to the data sets from the two locations Tübingen and Ulm. Using estimates of the genetic differentiation between each pair of populations (F_st_-values), the subprocedure MANTEL given in the programme *Genepop* was then used to test whether the genetic population structures in the location Ulm can be explained by an effect of isolation by distance.

Finally, to investigate temporal and spatial population structure we identified genetic clusters with the Bayesian model-based clustering algorithms implemented in *Structure* version 2.3.4 [39]–[41] under a model assuming admixture and correlated allele frequencies. Twenty runs with a burn-in period of 100,000 replications and a run length of 1,000,000 Markov chain Monte Carlo (MCMC) iterations were performed for a number of clusters ranging from K = 1 to 11. We first analysed the total data set including all populations from Tübingen and Ulm, and secondly the populations from Tübingen and Ulm were considered separately. Finally, we applied the *ad hoc* summary statistic ΔK developed by Evanno et al. 2005 [42] which is based on the rate of change of the ‘estimated likelihood’ between successive K values.

#### Graphical presentation of spatial and temporal population structures

The genetic distance between pairs of populations was estimated by Nei's (1987) [43] measure using the programme *POPDIST* version 1.2.0 [44]. The software *MEGA* version 5.1 [45] with the option UPGMA (unweighted pair group method with arithmetic mean) [46] was then applied to the genetic distance data in order to cluster the populations according to their pairwise genetic distances. Bootstrapping was performed for analysing the confidence of the topology. For this purpose we applied the programme *TreeFit* [47] with 100,000 repeats.

## Results

### Genetic variation of individual loci

#### Allelic diversity

Considering all loci, the number of alleles ranges from 2 to 8 in our sample over all years (Table S3 in File S1). The number of alleles at the individual loci and allelic richness was not associated with the size of the forest fragments (Table 3). Furthermore, the number of private alleles found in both study sites within the continuous forest (n = 8) was similar to that detected in the fragments (n = 9). Several loci were monomorphic for some study sites (locus Glis426 in HE_cont_ and HL_cont_, loci Glis426 and 11pilch in BS_frag_, locus Glis228 in SF_frag_, and the loci Glis196 and Glis228 in JH_frag_).

**Table 3:** The average degree of expected (*H*_e_) and observed (*H*_o_) heterozygosity of six populations in south western Germany; the means, the standard deviations and the effective number of alleles [*na* = 1/(1−*H*_e_)], number of private alleles and the allelic richness per study site are listed.

study site	*H*_e_ (*m* ± *s)*	*H*_o_ (*m* ± *s*)	*F_IS_*	*p/N*	*effective na*	*allelic richness*	*private alleles per population*
HE_cont_	0.409 ± 0.210	0.381 ± 0.208	0.028	0.114/146	1.692	2.558	2
HL_cont_	0.356 ± 0.225	0.367 ± 0.239	−0.078	0.965/48	1.553	2.349	0
BS_frag_	0.420 ± 0.286	0.408 ± 0.291	0.052	0.106/45	1.724	2.539	2
BG_frag_	0.388 ± 0.195	0.359 ± 0.202	0.094	**0.003**/71	1.634	2.601	1
JH_frag_	0.404 ± 0.250	0.351 ± 0.256	0.228	**0.009**/11	1.678	2.330	0
SF_frag_	0.402 ± 0.211	0.377 ± 0.201	0.072	0.051/51	1.672	2.449	2

Population specific F_IS_-values and their corresponding p-values with sample size N are given.

#### Genotypic variability and genotypic disequilibrium

The mean expected and observed degrees of heterozygosity were similar across all forest fragments (0.356≤*H*_e_≤0.420; 0.351≤*H*_o_≤0.408; Table 3). Testing for deviations of genotype frequencies from Hardy-Weinberg proportion and genotypic linkage disequilibria, the majority of tests did not indicate significance. As the few significant test results were not consistent with respect to loci, study sites and locations we assumed genetic equilibrium conditions (details see supporting information).

#### Temporal genetic variation

The temporal variation of allele frequencies within populations was studied by comparing the genetic structure between all pairs of years (test for allelic homogeneity). As samples from population HL_cont_ taken after 2003 were small, we did not consider these samples for homogeneity tests.

In all populations the allele frequencies were homogeneous in two successive years (across all loci, p≥0.396) but populations were genetically heterogeneous over longer time periods (HE_cont_: 2001 versus 2008 or 2009, p<0.001; BG_frag_ and SF_frag_: 2007 versus 2009, p « 0.001). The analyses of genotype variation between succeeding years yielded similar results (across all loci, p≥0.636). Three pairs of populations from different years showed significant differences after Bonferroni corrections (HE_cont_: 2001 versus 2008 and 2009, p<0.002; BG_frag_: 2007 versus 2009, p « 0.001).

Differences in the genetic variation among sites within the two locations Tübingen and Ulm were found. Dormice inhabiting the two sites in the continuous woodland (HE_cont_ and HL_cont_) were homogeneous in their allelic and genotypic structure across all loci in 2002 and 2003 (p≥0.175), and only in 2001 significant differences could be observed between the two study sites of the continuous forest (p « 0.001). In contrast, the fragmented and isolated populations around Ulm differed in their allelic and genotypic composition in all years between 2007 and 2009 (p<0.001).

### Genetic variability within and among populations

#### Source of molecular variation

We again removed the population of HL_cont_ from 2004 to 2009 in the genetic variance analyses due to their small sample sizes. Applying AMOVA to all populations studied in various years (Table S4 in File S1), the geographic isolation of the population in the continuous forest close to Tübingen and the forest fragments around Ulm becomes evident (*F*_CT_ = 0.131, p « 0.001). 13.05% of total genetic variation could be attributed to genetic differences between the two locations Tübingen and Ulm. The genetic differences among populations among years within one of the two locations contributed on average 6.41%. Finally, 4% of the total variation occurs by genetic differences among individuals within populations.

The large differences in the genetic structure between dormouse populations inhabiting the continuous forest and the forest fragments were best seen through the comparison of the results from the AMOVAs done separately for both locations (Table 4). The genetic structures differed significantly among subpopulations at both locations (Tübingen: *F*_CT_ = 0.015, p = 0.005; and Ulm: *F*_CT_ = 0.182, p<0.001). However, differentiation within the fragmented populations was 10 times higher than in the continuous woodland. Differences between both locations became also apparent in the parameter that reflects the stability of populations across years. The structures within the continuous forest at both sites HE_cont_ and HL_cont_ remained stable across 9 and 3 years, respectively (−0.13%, p = 0.697) whereas significant differences occurred in the populations of the forest fragments within 4 and 3 years, respectively (0.009%, p = 0.009). An effect of fragmentation might also be mirrored in the parameters measuring deviation from a random mating system: There was a significant higher genetic variation among individuals within populations living in the forest fragments (6.22%, p<0.001) than in the populations from the continuous forest (2.30%, p = 0.044).

**Table 4:** AMOVA of populations of the edible dormouse *Glis glis* at two locations (Tübingen and Ulm) in south western Germany.

source of variation	d.f.	sum of squares	variance components	variation explained (%)	F-statistics	significance (p)
**Tübingen**						
between HE_cont_ and HL_cont_	1	8.823	0.036	1.46	F_CT_ = 0.015	0.005
among yearly populations within HE_cont_ and HL_cont_	10	23.282	−0.003	−0.13	F_SC_ = −0.001	0.697
among individuals within populations	342	861.898	0.057	2.30	F_IS_ = 0.023	0.044
within individuals	354	851.500	2.405	96.37	F_IT_ = 0.036	0.042
Total	707	1,745.559	2.496			
**Ulm**						
among BS_frag_; JH_frag_; SF_frag_; BG_frag_	3	187.451	0.490	18.16	F_CT_ = 0.182	<0.001
among yearly populations within BS_frag_; JH_frag_; SF_frag_; BG_frag_	7	23.856	0.021	0.77	F_SC_ = 0.009	0.009
among individuals within populations	267	628.834	0.168	6.22	F_IS_ = 0.077	<0.001
within individuals	278	561.500	2.029	74.85	F_IT_ = 0.251	<0.001
Total	555	1,401.640	3.698			

The procedure AMOVA is separately applied to the populations of the two locations (Tübingen: HE_cont_ and HL_cont_ and Ulm: BS_frag_; JH_frag_; SF_frag_; BG_frag_). The results of the corresponding F-statistics are listed.

Finally, the genetic differences among local populations in Ulm cannot be explained by an effect of isolation by distance (MANTEL test; Spearman rank test p>0.248).

#### Population structure and admixture

**Figure 3:**
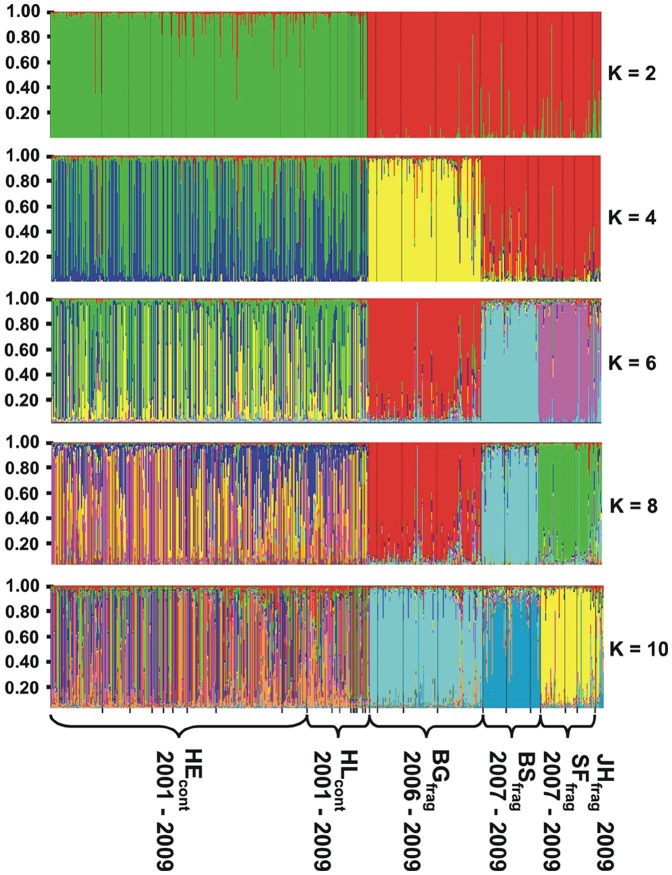
Assignment of dormouse individuals of six sites at two locations (Tübingen: HE_cont_ and HL_cont_; Ulm: BS_frag_; JH_frag_; SF_frag_; BG_frag_) in south western Germany to different source populations assuming different numbers of potential source populations (K = 2, 4, 6, 8, 10). Different colours indicate the possibility of different source populations. Colours in each column show the likelihood (%) to which source population an individual can be assigned.

**Figure 4:**
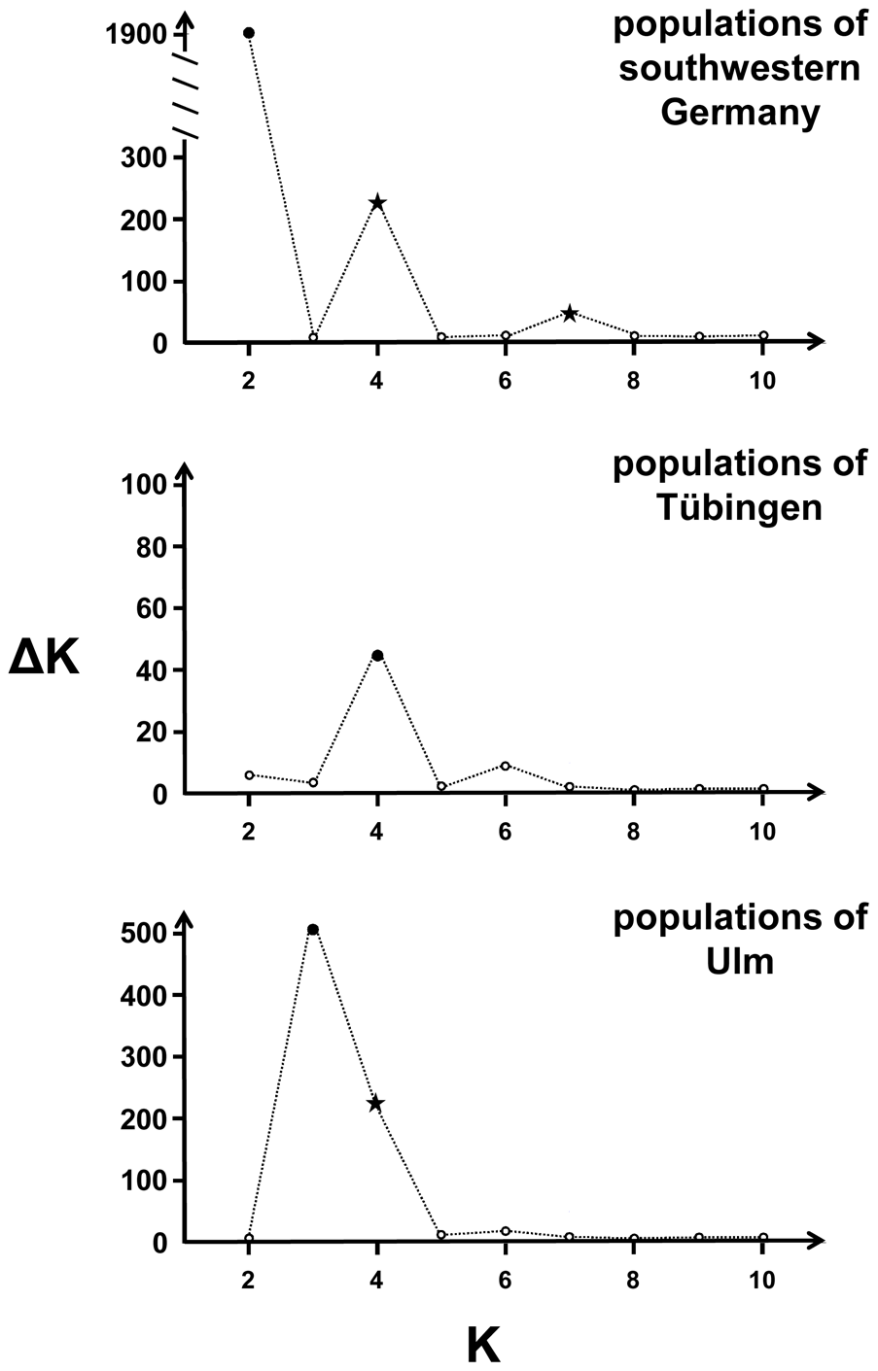
Graphical presentation of the Evanno et al.'s (2005) [**42**] statistics which estimates the most likely number of subpopulations explaining the genetic structure of populations. The maximum value of ΔK (filled dot) indicates the most probable number of populations (K). Additional local maxima are marked by asterisks.

The admixture model given in the programme *Structure* separates individuals from the study sites near Ulm from those close to Tübingen (Fig. 3). Assuming two source populations (K = 2), the programme assigns correctly all individuals to either Tübingen or Ulm (Fig. 3). Increasing the number of potential source populations (from K = 3 to K = 11), individuals are assigned to the forest fragments from which they were sampled. The ΔK-statistics identifies unequivocally the two main populations Ulm and Tübingen (ΔK = 1,925) but provides further evidence for some population substructuring (ΔK = 228 for K = 4 and ΔK = 50 for K = 7; Table S5 in File S1, Fig. 4). Indeed following a hierarchical analysis in testing separately the locations Tübingen and Ulm, we find that the individual genotypes from four populations living in forest fragments around Ulm can be assigned to at least three geographically distinct source populations (ΔK = 508, Fig. 4, Fig. S1). But some evidence is given that population structures might also be explained by four source populations (ΔK = 211, Fig. 4, Fig. S1). However, genotypes of individuals living in the continuous forest can be best explained by a mixture of dormice having an ancestral background of four different source populations (ΔK = 43, Fig. 4, Fig. S2) but here the test parameter ΔK is much lower than that of the location Ulm indicating less pronounced differentiation among subpopulations.

The design of the distribution patterns of our sample sites is not suitable to separate genetic structures resulting from isolation-by-distance from those of strict temporal isolation, i.e. the two sample sites in the continuous forest are close together and are not comparable to those of the fragmented forests. Nevertheless, our intention was to show that population structures between fragmented and continuous forests differ significantly, as they display highly homogeneous genetic structures in the fragmented forests whereas even closely neighboring populations of the continuous forest have a more heterogeneous background (Fig. 3 and Fig. S2).

### Graphical presentation of spatial and temporal population structures

**Figure 5:**
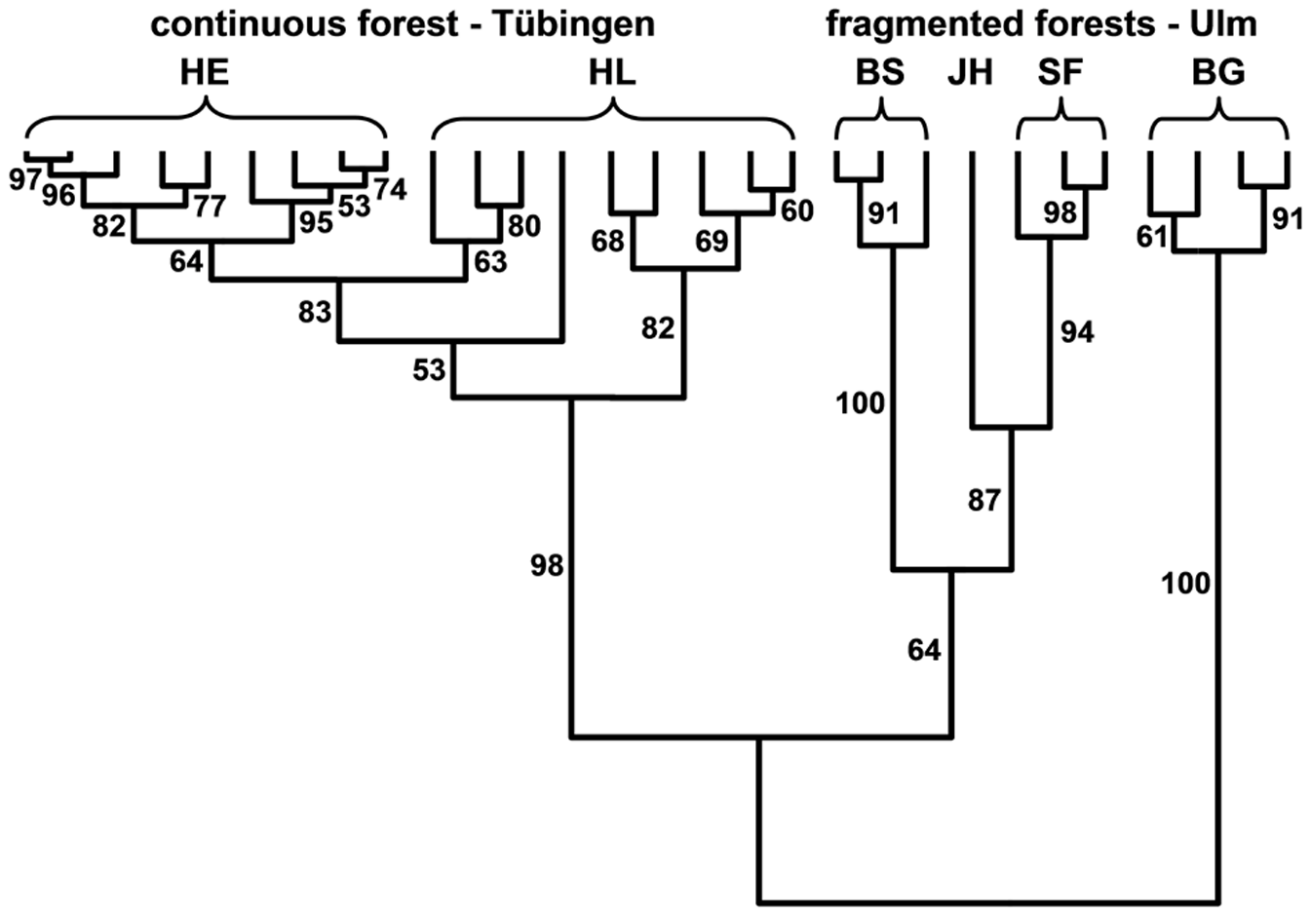
Clustering of edible dormouse populations from south western Germany using genetic distances between populations. Population structures were compared over a study period of several years (HE_cont_; HL_cont_ 2001–2009; BS_frag_ 2007–2009; BG_frag_ 2006–2009; JH_frag_ 2009; SF_frag_ 2007–2009). Genetic distances between populations were estimated by Nei's genetic distance [43] and grouped with the unweighted pair group method (UPGMA). Numbers indicate the reliability of nodes giving the bootstrap frequencies (%).

Dormice inhabiting the continuous forest (HE_cont_ and HL_cont_) formed a cluster of genetically highly similar populations over a period of 9 years (Fig. 5). The different populations in the forest fragments (BS_frag_; BG_frag_; JH_frag_; SF_frag_) were more distantly connected than the dormice inhabiting the two study sites in the continuous forest area (HE_cont_ and HL_cont_) but again the yearly samples grouped tightly together for each population. The bootstrapping procedure clearly supported the grouping of the Ulm fragments into distinct populations whereas the patterns of the populations from Tübingen did not indicate strong isolation of the two subpopulations (HE_cont_ and HL_cont_) over the total study period (0.53≤bootstrap≤1.00; R^2^ = 0.846).

## Discussion

### Isolation and genetic diversity

The arboreal edible dormouse has low dispersal abilities [20]; [21]; [22] and responses particularly sensitive to habitat fragmentation [9]; [14]; [15]; [16]; [17]. Results of our structure analyses now demonstrate that edible dormouse populations inhabiting forest fragments indeed represent genetically isolated populations. For such small and isolated populations the risk of extinction can rise through ecological factors like demographic or environmental stochastic events or/and through genetic effects as e.g. the loss of allelic diversity and inbreeding [7]; [48]; [49]. Lower genetic diversity in small and isolated populations compared to larger continuous populations has been observed in many species as for example the black grouse (*Tetrao tetrix*) [49], [50], the common hamster (*Cricetus cricetus*) [51] and forest habitat specialists like the arboreal common ringtail possums (*Pseudocheirus peregrinus*) and Australian squirrel gliders (*Petaurus norfolcensis*), which display lower levels of allelic richness and observed heterozygosity in small isolated populations than in populations inhabiting larger habitat patches [5]; [6].

Genetic variability in our study was very low in all dormouse populations (H_e_≤0.420 and H_o_≤0.408, Table 3). This phenomenon has also been found in other dormouse populations in Spain, Belgium, Poland and Germany [32]; [33]; [52]. The historical distribution of this species might explain low variability of microsatellite variation as a result of an overall low effective population size. Edible dormice have a strong preference for deciduous forests with a high proportion of seeding tree species like beeches (*F. sylvatica*) and oaks (*Quercus spec.*). It can therefore be assumed that during the glacial periods the geographical distribution of edible dormice with its low dispersal ability and strong habitat preference was restricted to the retreats of beeches and oaks in southern Europe. During the middle warm period (∼4,000 BC), however, dormice extended their distribution range throughout Central Europe following the expansion of beech dominated forests [8]. Consequently, the overall low genetic variability detected in edible dormice in Central Europe could be the result of a combination of several bottlenecks during the glacial periods and the invasion of only a few individuals that had colonized Central Europe after the last ice age.

### Genetic differentiation

Within Europe genetic studies on arboreal mammals investigating the connectivity among fragmented forest patches are scarce. One study on the red squirrel (*Sciurus vulgaris*) found high levels of gene flow across a comparably strongly fragmented landscape [53]. However, red squirrels are able to disperse comparatively long distances and pass open areas, using forest fragments as stepping stone patches. But even though gene flow of *S. vulgaris* was shown to be not disrupted by fragmentation, the red squirrel was rarely found in small forest fragments and is therefore believed to be especially sensitive to forest fragmentation [24].

In contrast to red squirrels, the dispersal ability of edible dormice is low [20]; [21]; [22], which results in reduced gene flow among local populations and in particular among populations inhabiting small forest fragments. Studying the effects of isolation and migration on population genetics, we also have to consider population sizes, as the genetic structure of small populations can assumed to be more affected by genetic drift and immigration than that of large populations [54]. In our south western dormouse populations, the number of individuals within the continuous forest (about 72,000 individuals, Table 1) exceeded by far the population sizes of the forest fragments (<2,000 individuals). These differences in population size can explain that the small populations differed in their allelic and genotypic structures among study years, whereas the large population of dormice (Table 1) inhabiting the continuous forest displayed only a low temporal variation (Fig. 5).

Genetic drift in small and isolated populations may lead to genetic differentiation of local populations which has been found for example in the common hamster in the Netherlands and Belgium [51]. In this context we assume that the genetic structure of small and isolated dormouse populations inhabiting forest fragments have also changed randomly and independently from each other (Fig. 5). Whereas the permanent exchange of individuals among neighbouring areas in large continuous populations might explain the low genetic differentiation between our two study sites in Tübingen. This scenario is supported by the cluster analysis which correctly assigns individuals from both sites of the continuous forest to a single large population consisting of a cluster of several subpopulations (K = 4, Fig. 4, Table S5 in File S1), whereas individuals from each of the isolated small forests were correctly assigned to one local population (Fig. 3 & 4, Table S5 in File S1). This clear result indicates that gene flow among populations is low and the genetic divergence of populations is increased [52]. Additional studies in other continuous and fragmented forest will help us to identify thresholds for such isolation processes. Further strong evidence of isolation is given by the presence of private alleles [54]. We detected nine alleles that were characteristic for the populations of the location Ulm. Seven of these alleles were not shared by all populations and five were private alleles occurring only in one of these populations (Table 3, Table S3 in File S1). Thus the isolation of fragmented forests, in which genetic drift acts and their temporal population dynamics might also differ due to, e.g., diseases, predators and catastrophes, can explain the genetic differences even among closely neighbouring populations (Fig. 1, Table 2). Furthermore, although we could not detect significant deviations of genotype frequencies from a Hardy-Weinberg proportion at individual loci, the F-statistics demonstrates a higher risk of inbreeding in populations inhabiting fragmented forests (F_IS_ = 0.077, p<0.001) than those in the continuous forest (F_IS_ = 0.023, p = 0.044), which can again support our assumption of a low number of immigrating individuals into the small forest fragments of the location Ulm.

### Conclusions

Edible dormice inhabiting forests fragments represent genetically isolated populations. Our finding of no significant differences in genetic diversity between forest fragments and the large continuous populations can possibly be explained by comparatively high population densities within the small forest fragments. However, we consider degrees of heterozygosity, allele numbers, and allelic richness as diversity measures to be rather insensitive when genetic variability of a species is generally low as shown for the edible dormouse. Nevertheless genetic isolation might be a precursor to genetic erosion and inbreeding depression to occur in the near future, which may limit or even prevent adaptive responses to environmental changes.

## Supporting Information

File S1 **Tables S1–S5.**
(RTF)

Figure S1 **Assignment of dormouse individuals of four sites at the location Ulm (BG_frag_ sampled from 2006 to 2009; BS_frag_ and SF_frag_ sampled from 2007 to 2009; JH_frag_ sampled in 2009) in south western Germany to different source populations assuming different numbers of potential source populations (K = 2, 3, 4, 5, 6).** Different colours indicate the possibility of different source populations. Colours in each column show the likelihood (%) to which source population an individual can be assigned.
(TIF)

Figure S2 **Assignment of dormouse individuals of two sites at the location Tübingen (HE_cont_** **and HL_cont_) in south western Germany to different source populations.** Samples from the population at site HE_cont_ was taken from 2001 to 2009, and from HL_cont_ from 2001 to 2003. For the assignment procedure we assumed different numbers of potential source populations (K = 2, 3, 4, 5, 6). Different colours indicate the possibility of different source populations. Colours in each column show the likelihood (%) to which source population an individual can be assigned.
(TIF)
