# Supplementary material for: Genetic Consequences of Forest Fragmentation for a Highly Specialized Arboreal Mammal - the Edible Dormouse
Source: PLoS One. 2014 Feb 4;9(2):e88092. doi: 10.1371/journal.pone.0088092 (PMC3913767; doi:10.1371/journal.pone.0088092)
Supplement: File S1 — Tables S1–S5. (RTF) [file pone.0088092.s001.rtf]

Supporting Information
Genotypic variability and heterozygosity
In HEcont the genotype frequencies at six loci showed significant deviations from Hardy-Weinberg proportions in some years. However, after Bonferroni correction with a total of 125 test repetitions, significance could only be found for the locus Glis173 in 2007 (p < 0.001).
Because of low sample sizes in 2004 to 2009 from the population HLcont, we did not apply Hardy- Weinberg tests to these samples. Nevertheless, in 2001 to 2003 the genotype distributions of all loci did not deviate significantly from Hardy-Weinberg proportions (p ≥ 0.119).
In the populations of the forest fragments 17 tests revealed significant deviations of the genotype frequency from a Hardy-Weinberg proportions. After Bonferroni correction with 135 test repetitions only three tests still yielded some significant results (BGfrag: Glis365 in 2009, 5pilch and 11pilch in 2007).

Genotypic linkage disequilibrium
In HEcont, only one out of 701 test results remained significant after the Bonferroni correction (Glis376- Glis487 in 2001, p « 0.001). Also in the population BGfrag one pair of loci (Glis365-5pilch in 2009) showed significant linkage disequilibrium applying the Bonferroni correction (p < 0.001; 354 pairs of loci tested). Furthermore, significant genotypic linkage disequilibrium (applying the Bonferroni correction) could not be observed in the population HLcont in 2001 to 2003 (197 pairs of loci tested, p ≥ 0.005), in BSfrag (177 pairs of loci tested, p ≥ 0.004), in JHfrag (66 pairs of loci tested, p ≥ 0.029) and in SFfrag (224 pairs of loci tested, p ≥ 0.006). The patterns of pairwise linkage were not consistent among populations and years and, therefore, all loci were considered independent.

Table S1: Sample sizes of adult individuals genotyped at 14 loci during the study period at six study sites in south western Germany (females/males) including recaptured individuals.
year	2001	2002	2003	2004	2005	2006	2007	2008	2009	total sample size	
Tübingen											
HEcont	33/28	17/15	16/10	9/5	6/5	10/7	17/17	38/40	14/15	160/142	
HLcont	15/15	3/6	6/7	0/3	0/2	0/2	1/6	1/2	1/4	27/47	
											
Ulm											
BSfrag							13/15	10/18	3/9	26/42	
BGfrag						5/5	15/15	25/16	31/22	76/58	
JHfrag									7/4	7/4	
SFfrag							14/15	6/8	13/9	33/32	

Table S2: 14 microsatellites studied in populations of the dormouse Glis glis. Genbank Accession Number, repeat motif and range of fragment length variation found in this study are given.
locus	accession number	repeat motif	fragment length (bp)	
Glis173	GU252362	CTAT	192-204	
Glis196	GU252363	TG	167-175	
Glis223	GU252364	GATA	150-178	
Glis228	GU252367	GATA/GAGA	142-150	
Glis239	GU252366	CTAT	131-155	
Glis243	GU252365	CTAT	271-279	
Glis365	GU252357	AGAT	194-214	
Glis376	GU252358	TATC	166-178	
Glis426	GU252359	AGAT	196-200	
Glis483	GU252360	TAGA	168-180	
Glis487	GU252361	GATA	170-182	
5pilch	FJ792767	CA	222-232	
8pilch	FJ792768	GGAA	279-287	
11pilch	FJ792764	CA	248-256	

Table S3: Allelic variation of edible dormice from 6 study sites in south western Germany. For each locus the fragment lengths of allelic PCR-products are given and the presence of alleles in a population is marked by a cross. Alleles observed only in the continuous forest or in the forest fragments are marked by bold capitals U (Ulm) or T (Tübingen). The total sample size over all years, and the expected and observed degrees of heterozygosity are listed. The degrees of heterozygosity are weighted means (sample sizes in years) of the yearly degrees of heterozygosity using the corresponding yearly sample sizes.
Glis173	192	196	200	204					N	He	Ho	
HEcont	X	X	X	X					299	0,434	0,318	
HLcont		X	X	X					74	0.252	0.284	
BSfrag		X	X	X					68	0.666	0.574	
Ulm. BGfrag		X	X	X					114	0.503	0.377	
JHfrag	X	X	X	X					11	0.606	0.455	
SFfrag	X	X	X	X					65	0.349	0.292	
Glis 196	167	169	171	173	175							
HEcont	X	X	X	X	X				301	0.326	0.311	
HLcont	X	X	X		X				74	0.252	0.265	
BSfrag		X		X	X				68	0.127	0.133	
BGfrag	X	X	X	X					129	0.391	0.248	
JHfrag			X						10	0.000	0.000	
SFfrag	X		X						65	0.046	0.046	
Glis 223	150	154	158	162	166	170	174	178				


HEcont	X	X	X	X	T	T	T	T	300	0.769	0.760	
HLcont	X	X	X	X	T	T	T		74	0.741	0.676	
BSfrag	X	X	X	X					68	0.634	0.765	
BGfrag	X	X	X	X					134	0.217	0.239	
JHfrag		X	X	X					11	0.675	0.545	
SFfrag	X	X	X	X					65	0.662	0.523	
Glis228	142	146	150									
HEcont	T	X	X						299	0.404	0.372	
HLcont	T	X	X						74	0.365	0.392	
BSfrag		X	X						68	0.199	0.221	
BGfrag		X	X						131	0.274	0.321	
JHfrag			X						11	0.000	0.000	
SFfrag			X						65	0.000	0.000	
Glis239	131	135	139	143	147	151		155				
HEcont		T		X	X	X		X	301	0.553	0.575	
HLcont				X	X	X			74	0.563	0.583	
BSfrag	U		U	X	X	X		X	68	0.645	0.588	
BGfrag			U	X	X	X		X	43	0.498	0.512	
JHfrag			U	X	X	X			10	0.611	0.800	
SFfrag			U	X	X	X		X	65	0.688	0.615	


Glis243	271	275	279	283								
HEcont	X	X	X	T					296	0.541	0.544	
HLcont	X	X	X						74	0.541	0.678	
BSfrag	X	X	X						68	0.617	0.573	
BGfrag	X	X	X						134	0.542	0.508	
JHfrag	X	X	X						11	0.515	0.364	
SFfrag	X	X	X						65	0.595	0.615	
Glis365	194	198	202	206	210	214						
HEcont		X	X	X	X	X			299	0.448	0.402	
HLcont		X	X	X	X				74	0.545	0.554	
BSfrag	U	X	X	X	X				68	0.713	0.750	
BGfrag	U	X	X	X	X	X			134	0.747	0.724	
JHfrag	U	X	X	X					11	0.610	0.364	
SFfrag	U	X	X	X					65	0.582	0.492	
Glis376	166	170	174	178								
HEcont	X	X	X	X					296	0.448	0.402	
HLcont		X	X	X					74	0.347	0.243	
BSfrag	X	X	X	X					68	0.666	0.529	
BGfrag		X	X	X					131	0.372	0.351	
JHfrag		X	X	X					11	0.437	0.364	


SFfrag		X	X	X					65	0.232	0.170	
Glis426	196	200										
HEcont	X								236	0.000	0.000	
HLcont	X								60	0.000	0.000	
BSfrag	X								68	0.000	0.000	
BGfrag	X	U							121	0.269	0.206	
JHfrag	X	U							11	0.091	0.091	
SFfrag	X	U							65	0.361	0.370	
Glis483	168	172	176	180								
HEcont	X	X	X						152	0.586	0.454	
HLcont	X	X	X						27	0.560	0.556	
BSfrag	X	X	X	U					26	0.535	0.654	
BGfrag	X	X	X						76	0.508	0.618	
JHfrag		X	X						7	0.527	0.571	
SFfrag		X	X						33	0.470	0.515	
Glis487	170	174	178	182								
HEcont		X	X	X					302	0.193	0.149	
HLcont		X		X					74	0.040	0.040	
BSfrag		X	X						68	0.086	0.088	
BGfrag		X	X	X					132	0.075	0.076	


JHfrag	U	X	X						11	0.178	0.182	
SFfrag		X	X						64	0.258	0.297	
5pilch	222	224	226	228	230	232						
HEcont			X	X	X	X			294	0.504	0.531	
HLcont			X	X	X				74	0.545	0.635	
BSfrag		U	X	X	X	X			68	0.712	0.691	
BGfrag		U	X	X	X	X			131	0.660	0.595	
JHfrag	U			X	X	X			11	0.723	0.727	
SFfrag				X	X	X			65	0.474	0.538	
8pilch	279	283	287									
HEcont	X	X	T						289	0.104	0.107	
HLcont	X	X	T						74	0.130	0.108	
BSfrag	X	X							68	0.255	0.147	
BGfrag	X	X							126	0.181	0.198	
JHfrag	X	X							11	0.368	0.091	
SFfrag	X	X							65	0.444	0.308	
11pilch	250	254	258									
HEcont		X	X						300	0.276	0.267	
HLcont		X	X						74	0.356	0.367	
BSfrag		X							68	0.000	0.000	


BGfrag	U	X	X						130	0.211	0.93	
JHfrag		X	X						11	0.404	0.351	
SFfrag		X	X						65	0.470	0.508	

Table S4: AMOVA of populations of the edible dormouse (Glis glis) studied in six sites at two locations (Tübingen: HEcont and HLcont; Ulm: BSfrag; JHfrag; SFfrag; BGfrag) in southwestern Germany. The results of the corresponding F-statistics and significance values are listed.
source of variation	d.f.		sum of squares	variance components	proportion of variation (%)	F-statistics	significance (p)	
between locations		1	231.410	0.348	13.05	FCT = 0.131	« 0.001	
among yearly populations within locations		21	237.610	0.171	6.41	FSC = 0.074	« 0.001	
among individuals within populations		609	1,371.984	0.106	3.99	FIS = 0.050	« 0.001	
within individuals		632	1,289.500	2.038	76.56	FIT = 0.234	« 0.001	
Total	1,263	3,130.504	2.657				

Table S5: Analysis of the assignment simulations (programme Structure version 2.3.4, [39-41]) with the ad hoc summary statistic ÄK developed by Evanno et al. (2005) [42]. The parameters resulting from 20 simulations (the mean of L(K), the standard deviation of L(K) s[L(K)], L'(K), L''(K) and ÄK) are listed given the number of assumed populations K = 1 to 11 (for more detail see [42]).
K	L(K)	s[L(K)]	L'(K)= 
L(K)-L(K-1)	L''(K)= ²L'(K+1)-L'(K)²	ÄK= L''(K)/s[L(K)]	
    populations of southwestern Germany	
1	-15,241.315	0.037				
2	-13,625.770	0.604	1,615.545	1,164.605	1,925.041	
3	-13,173.830	168.921	451.940	58.200	0.345	
4	-12,780.090	1.068	393.740	243.235	227.716	
5	-12,629.585	62.766	150.505	72.625	1.157	
6	-12,406.455	60.962	223.130	28.145	0.462	
7	-12,211.470	2.358	194.985	118.450	50.243	
8	-12,134.935	14.904	76.535	3.505	0.235	
9	-12,054.895	47.101	80.040	12.095	0.257	
10	-11,986.950	50.997	67.945	48.770	0.956	
11	-11,967.775	120.656	19.175			
     populations of Tübingen	
  1	          -7014,714	    0.035				
  2	    -6810.488	  18.009	  204.226	  81.072	4.502	
  3	    -6687.334	  13.571	123.154	  35.098	2.660	
  4	    -6528.082	1.466	159.252	  63.494	43.318	
  5	    -6432.324	2.780	95.758	5.630	2.025	
  6	    -6330.936	3.391	101.388	33.430	9.858	
  7	    -6262.978	25.040	67.958	41.702	1.665	
  8	    -6236.722	42.429	26.256	7.882	0.186	
  9	    -6202.584	48.252	34.138	3.654	0.076	
10	    -6164.792	66.837	37.792	5.664	0.085	
11	     -6121.336	11.961				


populations of Ulm
-11,967.775
120.656
19.175	
  1	          -6238.948	128.499				
  2	    -5656.270	182.770	  582.678	263.800	1.443	
  3	    -5337.392	   0.342	318.878	173.370	507.569	
  4	    -5191.884	0.480	145.508	101.496	211.285	
  5	    -5147.872	21.826	44.012	36.240	1.660	
  6	    -5067.620	4.812	80.252	29.610	6.154	
  7	    -5016.978	20.569	50.642	21.824	1.061	
  8	    -4944.512	10.402	72.466	5.238	0.504	
  9	    -4877.284	29.168	24.984	42.244	1.448	
10	    -4852.300	27.549	37.792	4.012	0.146	
11	     -4823.304	10.573	28.996			
