# Supplementary figures and images for: Genetic Consequences of Forest Fragmentation for a Highly Specialized Arboreal Mammal - the Edible Dormouse
Source: PLoS One. 2014 Feb 4;9(2):e88092. doi: 10.1371/journal.pone.0088092 (PMC3913767; doi:10.1371/journal.pone.0088092)

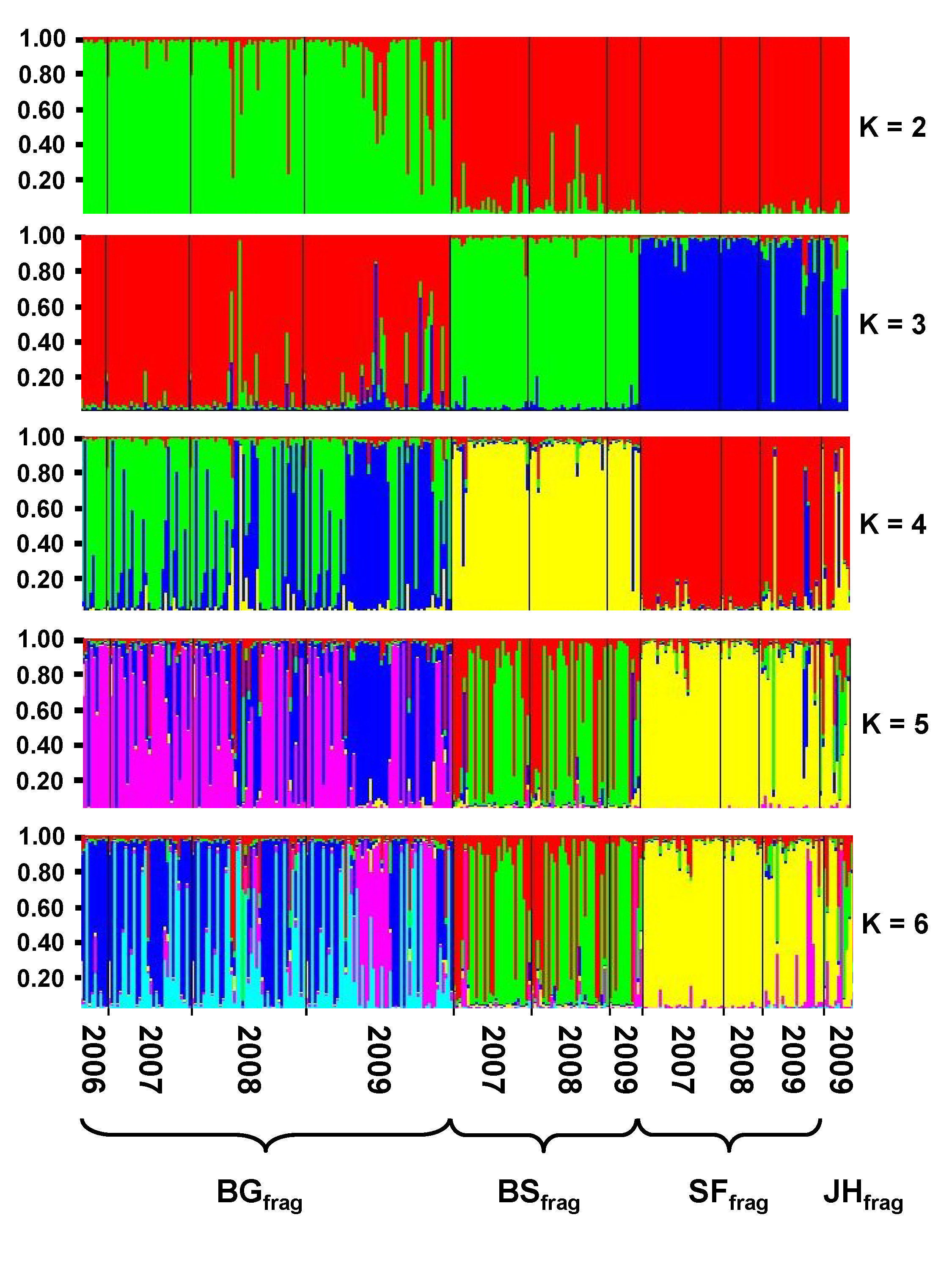

Supplement: Figure S1 — Assignment of dormouse individuals of four sites at the location Ulm (BGfrag sampled from 2006 to 2009; BSfrag and SFfrag sampled from 2007 to 2009; JHfrag sampled in 2009) in south western Germany to different source populations assuming different numbers of potential source populations (K = 2, 3, 4, 5, 6). Different colours indicate the possibility of different source populations. Colours in each column show the likelihood (%) to which source population an individual can be assigned. (TIF) [file pone.0088092.s002.tif]

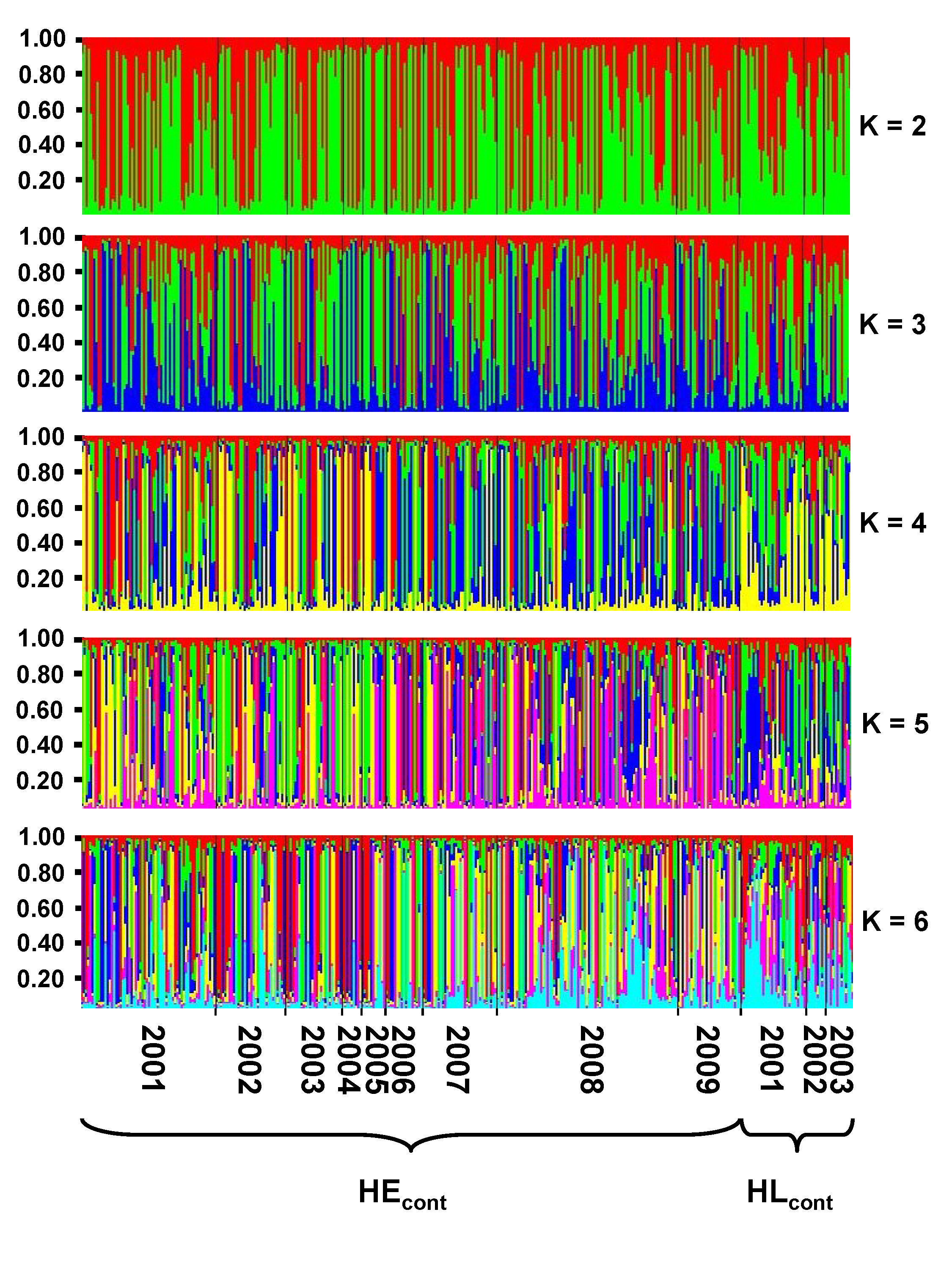

Supplement: Figure S2 — Assignment of dormouse individuals of two sites at the location Tübingen (HEcont and HLcont) in south western Germany to different source populations. Samples from the population at site HEcont was taken from 2001 to 2009, and from HLcont from 2001 to 2003. For the assignment procedure we assumed different numbers of potential source populations (K = 2, 3, 4, 5, 6). Different colours indicate the possibility of different source populations. Colours in each column show the likelihood (%) to which source population an individual can be assigned. (TIF) [file pone.0088092.s003.tif]
